# Cerium (IV) Ammonium Nitrate (CAN) Catalyzed One-pot Synthesis of 2-Arylbenzothiazoles 

**DOI:** 10.3390/molecules13112908

**Published:** 2008-11-24

**Authors:** Fawzia Al-Qalaf, Ramadan Ahmed Mekheimer, Kamal Usef Sadek

**Affiliations:** 1 Applied Science Department, College of Technological Sudies, Public Authority for Applied Education and Training, Safat 13060, Kuwait; 2 Chemistry Department, Faculty of Science, El-Minia University, El-Minia 61519, A. R. Egypt E-mail: rmekh@yahoo.com (R-A. M.)

**Keywords:** One-pot synthesis, 2-Arylbenzothiazoles, CAN.

## Abstract

A one-pot synthesis of 2-arylbenzothiazoles from the reaction of 2-amino-thiophenol and aromatic aldehydes catalysed by cerium (IV) ammonium nitrate (CAN) is reported.

## Introduction

2-Arylbenzothiazoles are a class of molecules which posses an interesting variety of biological activities [1,2,3]. They are a class of potent and selective antitumor agents which exhibit nanomolar inhibitory activity against a range of human breast, ovarian, colon and renal cell lines *in vitro* [4]. In addition, they represent one of the most promising antiamyloid therapies for treatment of a number of a heterogenous family of diseases referred to generically as amyloidosis, including Alzheimer’s disease (AD), type II diabetes, variant Creutzfeldt-Jakob disease, painful joints associated with long term hemodialysis and rare cases of hereditary insomnia [5,6]. 

In general, benzothiazoles are synthesized by condensation of 2-aminothiophenol with carboxylic acid derivatives [7], the base induced cyclization of the corresponding 2-haloanilides [8], or the radical cyclization of thioacylbenzanilides [9]. On the other hand, the most general synthetic approaches to 2-arylbenzothiazoles involve: (1) arylation of benzothiazole with aryl bromides at 150^o^C in a sealed tube catalyzed by Pd(OAc)_2_, Cs_2_CO_3_ and CuBr with P(*t*-Bu)_3_ as ligand [10], or Suziki biaryl coupling of 2‑bromobenzothiazole with aryl boronic acids [11]; (2) condensation of 2-aminothiophenols with carboxylic acids under microwave irradiation [12] or with polymer-bound esters in the presence of a Lewis acid [13]; (3) oxidative cyclization of phenolic Schiff’s bases derived from the condensation of 2-aminothiophenols and aldehydes using various oxidants such as Sc(OTf)_3_ using molecular oxygen[14], activated carbon [15], pyridinium chlorochromate [16] and very recently *via* electrooxidation [17]; recently [18] a modification of such strategy that involves flash vacuum pyrolysis and photolysis of 2-methylthio-*N*-(arenylidene)anilines has been reported; (4) direct condensation of 2-aminothiophenol with aromatic aldehydes under microwave irradiation [19]. However, most of these synthetic approaches suffer from drawbacks such as harsh reaction conditions (strong acids, high temperatures), lengthy procedures that consume excess reagents, expensive catalysts that may be harmful to the environment or a sophisticated techniques.

Cerium (IV) ammonium nitrate (CAN) is one of the most interesting oxidants in organic synthesis since it is stable in different solvents and is commercially available. The use of this reagent for numerous reactions involving C-S, C-N, C-Se and C-Cl bond formation has been reviewed [20,21,22]. In continuation of our work aiming at development of efficient and simple techniques for the synthesis of heterocycles [23,24], we reported herein a one-pot synthesis of 2-arylbenzothiazole from the reaction of 2-aminothiophenol **1** with aromatic aldehydes **2** catalyzed by CAN.

## Results and Discussion

Reaction of equimolar amounts of **1** and **2** in methanol in the presence of CAN produces the corresponding 2-arylbenzothiazoles **4** in good yields (75-89%) *via* the intermediacy of Schiff’s bases **3**.

**Figure 1:**
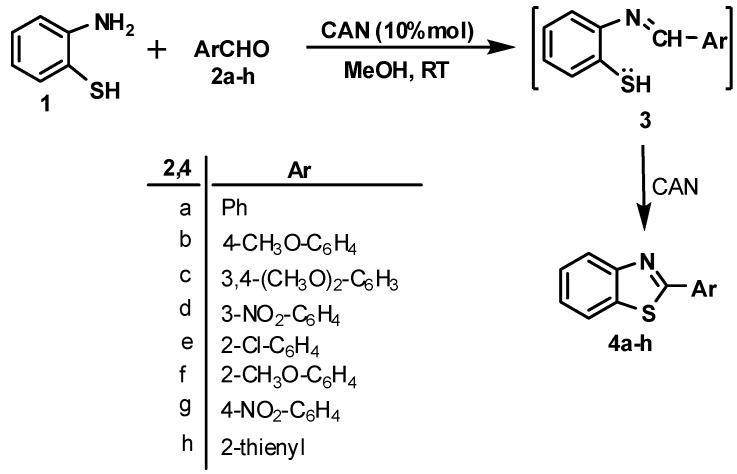
One-pot synthesis of 2-arylbenzothiazoles **4a-h.**

**Figure 2:**
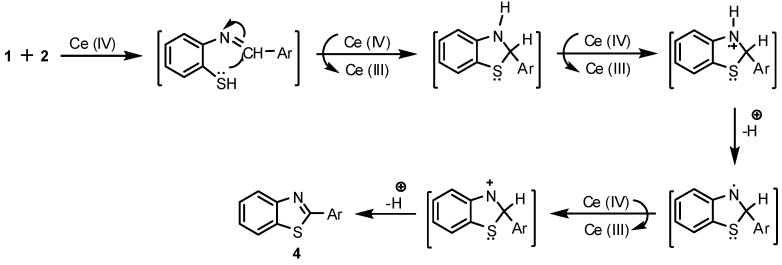
A proposed mechanism for the formation of **4a-h.**

In order to investigate the optimum conditions for these reactions we first studied the efficacy of the ratio of the catalyst (2, 5, 10, 15 mol %) and our study revealed that 10 mol% of the catalyst was the optimium ratio. For 2 and 5 mol% of CAN, the yields decreased to 55-62% and 63-73%, respectively, whereas an increase in the quantity of CAN has no significant effect on the overall yield. In addition, methanol was the best solvent among those tested (H_2_O, acetone, CHCl_3_). Next we studied the effect of aromatic aldehyde substituent on the reaction rate and the overall yield. With both electron withdrawing and electron donating groups the reaction proceeds smoothly, with a slight increase in the yield when the aryl substituent was an electron withdrawing group. A mechanism to account for the formation of **4a-h** is proposed in Figure 2. It is clearly obvious that CAN acts as both a Lewis acid and an oxidant.

## Conclusions

Cerium (IV) ammonium nitrate (CAN) has been employed for the first time as a mild and efficient reagent for the one-pot synthesis of 2-arylbenzothiazoles in high yields. The procedure proved to be simple either in conducting the reaction or isolation of the products and to the best of our knowledge it is one of the few reported direct one-pot synthesis of 2-arylbenzothiazoles from the reaction of 2-aminothiophenol with aromatic aldehydes and at ambient temperature.

## Experimental

### General

Melting points were determined on a Gallenkamp melting point apparatus and are uncorrected. Infrared spectra were measured with a Shimadzu Model 470 spectrophotometer. The NMR spectra were recorded on a Bruker AM 400 spectrometer with DMSO-*d*_6_ as solvent and TMS as internal reference, chemical shifts are expressed as *δ* ppm. Mass spectra were measured on a GCMS-QP1000EX mass spectrometer. Analytical data were determined on the Microanalytical Data Unit at Kuwait University. Analytical TLC was performed with silica gel plates using silica gel 60 PF_254_ (Merck).

### Synthesis of 2-arylbenzothiazoles ***4a-h***

To a mixture of **1** (0.626 g, 5 mmol) and the appropriate aldehyde **2** (5 mmol) in methanol (10 mL) was added with stirring 10% mol of cerium (IV) ammonium nitrate (CAN). The reaction mixture was stirred at room temperature overnight. Brine solution was then added to the mixture and the solid formed was collected by filtration, dried and recrystallised from EtOH to afford compounds **4a-h**.

*2-Phenyl-1,3-benzothiazole* (**4a**): Yield: 75%; m.p. 112-113˚C (Lit.^25^: 115-116^o^C); IR (*ν*_max_, KBr, cm^‑1^): 3060, 3018, 1609, 1585; ^1^H-NMR: δ 7.48 (t, *J* = 7.8 Hz, 1H, Ar-H), 7.54-7.60 (m, 4H, Ar-H), 8.07-8.12 (m, 3H, Ar-H), 8.16 (d, *J* = 7.8 Hz, 1H, Ar-H); MS m/z (rel. int. %) 211 (M^+^, 100); Anal. calcd. for C_13_H_9_NS: C, 73.90; H, 4.29; N, 6.63; S, 15.17. Found: C, 73.79; H, 4.19; N, 6.81; S, 14.98.

*2-(4-Methoxyphenyl)-1,3-benzothiazole* (**4b**): Yield: 78%; m.p.126-128˚C (Lit. [17]: 119-120^o^C); IR (*ν*_max_, KBr, cm^-1^): 3103, 3058, 1604, 1585; ^1^H-NMR: δ 3.86 (s, 3H, OCH_3_), 7.12 (d, *J* = 7.8 Hz, 2H, Ar-H), 7.43 (t, *J* = 7.8 Hz, 1H, Ar-H), 7.53 (t, *J* = 8.0 Hz, 1H, Ar-H), 8.01-8.10 (m, 3H, Ar-H), 8.11 (d, *J* = 7.6 Hz, 1H, Ar-H); MS m/z (rel. int. %) 241 (M^+^, 100); Anal. calcd. for C_14_H_11_NOS: C, 69.68; H, 4.59; N, 5.80; S, 13.29. Found: C, 69.55; H, 4.52; N, 5.94; S, 13.18.

*2-(3,4-Dimethoxyphenyl)-1,3-benzothiazole* (**4c**): Yield: 77%; m.p.130-132˚C; IR (*ν*_max_, KBr, cm^-1^): 3078, 3053, 2962, 2835, 1600; ^1^H-NMR: δ 3.85 (s, 3H, OCH_3_), 3.89 (s, 3H, OCH_3_), 7.11 (d, *J* = 8.4 Hz, 1H, Ar-H), 7.41 (t, *J* = 7.8 Hz, 1H, Ar-H), 7.53 (t, *J* = 7.8 Hz, 1H, Ar-H), 7.60-7.66 (m, 2H, 2Ar-H), 8.02-8.09 (m, 1H, Ar-H), 8.10 (d, *J* = 8.4 Hz, 1H, Ar-H); MS m/z (rel. int. %) 271 (M^+^, 100); Anal. calcd. for C_15_H_13_NO_2_S: C, 66.40; H, 4.83; N, 5.16; S, 11.82. Found: C, 66.33; H, 4.72; N, 5.22; S, 11.69.

*2-(3-Nitrophenyl)-1,3-benzothiazole* (**4d**): Yield: 88%; m.p. 184-186˚C (Lit. [16]: 181-183^o^C); IR (*ν*_max_, KBr, cm^-1^): 3080, 3033, 1612, 1577; ^1^H-NMR: δ 7.54 (t, *J* = 8.0 Hz, 1H, Ar-H), 7.60 (t, *J* = 8.0 Hz, 1H, Ar-H), 7.88 (t, *J* = 8.0 Hz, 1H, Ar-H), 8.16 (d, *J* = 8.0 Hz, 1H, Ar-H), 8.24 (d, *J* = 8.0 Hz, 1H, Ar-H), 8.41 (d, *J* = 8.0 Hz, 1H, Ar-H), 8.44 (d, *J* = 8.0 Hz, 1H, Ar-H), 8.84 (s, 1H, Ar-H); ^13^C-NMR: δ_C_ 111.8, 119.5, 120.3, 122.5, 126.3, 126.5, 130.2, 130.7, 133.3, 142.8, 149.4, 157.5, 161.9; MS m/z (rel. int. %) 256.0 (M^+^, 100); Anal. calcd. for C_13_H_8_N_2_O_2_S: C, 60.93; H, 3.15; N, 10.93; S, 12.51. Found: C, 60.86; H, 3.27; N, 11.02; S, 12.64.

*2-(2-Chlorophenyl)-1,3-benzothiazole* (**4e**): Yield: 89%; m.p. 71-73˚C (Lit. [16]: 71-73^o^C); IR (*ν*_max_, KBr, cm^-1^): 3050, 3035, 1559; ^1^H-NMR: δ 7.33-7.44 (m, 3H, Ar-H), 7.51-7.54 (m, 2H, Ar-H), 7.93 (d, *J* = 7.6 Hz, 1H, Ar-H), 8.12 (d, *J* = 8.0 Hz, 1H, Ar-H), 8.21-8.25 (m, 1H, Ar-H); ^13^C-NMR: δ_C_ 121.4, 123.6, 125.6, 126.4, 127.2, 130.9, 131.3, 131.9, 132.4, 132.8, 136.2, 152.6, 164.3; Anal. calcd. for C_13_H_8_ClNS: C, 63.54; H, 3.28; N, 5.70. Found: C, 63.45; H, 3.41; N, 5.76.

*2-(2-Methoxyphenyl)-1,3-benzothiazole* (**4f**): Yield 80%; m.p.102-103^o^C (Lit. [19]: 101-103^o^C); IR: (*ν*_max_, KBr, cm^-1^): 3105, 3050, 1605, 1588; ^1^H-NMR: δ 3.83 (s, 3H, OCH_3_); 7.03 (d, *J*= 7.8 Hz, 1H, Ar-H); 7.15 (t, *J* = 7.8 Hz, 1H, Ar-H); 7.40-7.53 (m, 2H, Ar-H), 7.68 (d, *J* = 7.8 Hz, 1H, Ar-H), 8.01 (d, *J* = 7.6 Hz, 1H, Ar-H); 8.18 (d, *J* = 7.8 Hz, 1H, Ar-H); MS m/z (rel. int. %) 241 (M^+^, 100); Anal. calcd. for C_14_H_11_NOS: C, 69.68; H, 4.59; N, 5.80; S, 13.29. Found: C, 69.52; H, 4.55; N, 5.93; S, 13.22.

*2-(4-Nitrophenyl)-1,3-benzothiazole* (**4g**): Yield 87%; m.p. 224-225^o^C (Lit. [17]: 224-226^o^C): IR (*ν*_max_, KBr, cm^-1^): 3082, 3035, 1615, 1580; ^1^H-NMR: δ 7.44-7.53 (m, 2H, Ar-H) ; 8.01 (d, *J* = 8.0 Hz, 1H, Ar-H); 8.10 (d, *J* = 8.0 Hz, 2H, Ar-H); 8.21 (d, *J* = 8.0 Hz, 1H, Ar-H); 8.32 (d, *J* = 8.0 Hz, 2H, Ar-H); MS m/z (rel. int. %) 256.0 (M^+^, 100); Anal. calcd. for C_13_H_8_N_2_O_2_S: C, 60.93; H, 3.15; N, 10.93; S, 12.51. Found: C, 60.88; H, 3.25; N, 11.05; S, 12.64.

*2-Thienyl-1,3-benzothiazole* (**4h**): Yield 79%; m.p. 97-99^o^C (Lit. [26]: 98-100^o^C): IR (*ν*_max_, KBr, cm^‑1^): 3080, 3040, 1620; ^1^H-NMR: δ 7.31 (t, *J* = 4.0 Hz, 1H, thiophene CH); 7.54-7.63 (m, 2H, Ar-H); 7.69 (d, *J* = 4.0 Hz, 1H, thiophene CH); 7.72 (d, *J* = 4.0 Hz, 1H, thiophene CH); 8.14 (d, *J* = 8.0 Hz, 1H, Ar-H), 8.22 (d, *J* = 8.0 Hz, 1H, Ar-H); MS m/z (rel. int. %) 217.0 (M^+^, 100); Anal. calcd. for C_11_H_7_NS_2_: C, 60.82; H, 3.22; N, 6.44; S, 29.52. Found: C, 60.73; H, 3.28; N, 6.62; S, 29.34.
